# Towards sex identification of Asian Palmyra palm (*Borassus flabellifer* L.) by DNA fingerprinting, suppression subtractive hybridization and *de novo* transcriptome sequencing

**DOI:** 10.7717/peerj.7268

**Published:** 2019-07-10

**Authors:** Kwanjai Pipatchartlearnwong, Piyada Juntawong, Passorn Wonnapinij, Somsak Apisitwanich, Supachai Vuttipongchaikij

**Affiliations:** 1 Department of Genetics, Faculty of Science, Kasetsart University, Bangkok, Thailand; 2 Center of Advanced studies for Tropical Natural Resources, Kasetsart University, Bangkok, Thailand; 3 Omics Center for Agriculture, Bioresources, Food and Health, Kasetsart University (OmiKU), Bangkok, Thailand

**Keywords:** Sex-linked marker, Palm-sugar, Dioecious, DNA marker, Arecaceae

## Abstract

**Background:**

Asian Palmyra palm, the source of palm-sugar, is dioecious with a long juvenile period requiring at least 12 years to reach its maturity. To date, there is no reliable molecular marker for identifying sexes before the first bloom, limiting crop designs and utilization. We aimed to identify sex-linked markers for this palm using PCR-based DNA fingerprinting, suppression subtractive hybridization (SSH) and transcriptome sequencing.

**Methods:**

DNA fingerprints were generated between males and females based on RAPD, AFLP, SCoT, modified SCoT, ILP, and SSR techniques. Large-scale cloning and screening of SSH libraries and *de novo* transcriptome sequencing of male and female cDNA from inflorescences were performed to identify sex-specific genes for developing sex-linked markers.

**Results:**

Through extensive screening and re-testing of the DNA fingerprints (up to 1,204 primer pairs) and transcripts from SSH (>10,000 clones) and transcriptome data, however, no sex-linked marker was identified. Although *de novo* transcriptome sequencing of male and female inflorescences provided ∼32 million reads and 187,083 assembled transcripts, PCR analysis of selected sex-highly represented transcripts did not yield any sex-linked marker. This result may suggest the complexity and small sex-determining region of the Asian Palmyra palm. To this end, we provide the first global transcripts of male and female inflorescences of Asian Palmyra palm. Interestingly, sequence annotation revealed a large proportion of transcripts related to sucrose metabolism, which corresponds to the sucrose-rich sap produced in the inflorescences, and these transcripts will be useful for further understanding of sucrose production in sugar crop plants. Provided lists of sex-specific and differential-expressed transcripts would be beneficial to the further study of sexual development and sex-linked markers in palms and related species.

## Introduction

Asian Palmyra palm (*Borassus flabellifer* L, 2*n* = 36) is a dioecious and slow-growing perennial tree, requiring 12–15 years to reach its maturity and produce the first inflorescence (Kovoor, 1983). Once flowered, it vigorously and continuously produces flowers and fruits through its lifespan. This palm is found widespread in South and Southeast Asia and provides essential food and economic values throughout its parts including inflorescence sap for producing palm sugar and alcoholic beverages, fruits for consumption and the tree trunk for construction (Morton, 1988; Lim, 2012). Both sexes are morphologically identical except for the male and female inflorescences, and there are no reliable means of sex identification before the first bloom. Because of the long juvenile period and a preference for female plants for fruit production, growers hesitate to expand the plantation, and this limits the utilization of this palm. Being able to identify the sexes of Asian Palmyra palm at seedling stages will provide better designs for the crop production through optimal male and female ratios, breeding programs, conservation and utilization (Davis & Johnson, 1987; Barfod et al., 2015; Sirajuddin et al., 2016). Molecular markers for sex identification in Asian Palmyra palm is needed.

Asian Palmyra palm belongs to family Arecaceae, which includes many palm species that are among the world commercially important crops, including oil palm (*Elaeis oleifera*), coconut (*Cocos nucifera*) and date palm (*Phoenix dactylifera*) (Beck & Balick, 1990; Dransfield et al., 2005). Historically, it was placed among these valued crops as for producing palm sugar, alcohol products and its nutritious fruits dating back at least 2,500 years (Fox, 1977; Ferguson, 1888), before sugarcane gradually replaced its status. Historical and genetic studies suggest that Asian Palmyra palm was originated from tropical Africa, brought along the spice route to the east and settled in the Indian subcontinent, where it is widely propagated throughout India and Sri Lanka, before introduced to Southeast Asia more than 1,500 years ago (Pipatchartlearnwong et al., 2017a). Given its extended benefits since the historical time and because of its vigorous growth and continuous supply of inflorescence sap and fruits with the ability to withstand severe climate, arid conditions, pests and diseases, Asian Palmyra palm should be recognized as a potential recalcitrant food crop to the climate change for the tropical and subtropical regions.

Molecular data of Asian Palmyra palm are currently limited. The genome sequence is not available, and only its chloroplast genome was recently reported (Sakulsathaporn et al., 2017). A few sex-linked markers have been developed based on DNA fingerprinting, but only one RAPD-based marker was shown to be able to identify the sexes among the populations in India (George et al., 2007). However, our preliminary experiment showed that this marker was unable to identify the sexes in the population in Thailand. Previously, we showed that Asian Palmyra palm populations in Thailand are descended from a small number of seedlings brought in at least 1,500 years ago and represent a very narrow genetic diversity (Pipatchartlearnwong et al., 2017a; Pipatchartlearnwong et al., 2017b).

In this work, we aimed to develop sex-linked markers of Asian Palmyra palm through three approaches: DNA fingerprinting, direct cloning of subtraction subtractive hybridization (SSH) of cDNA from male and female inflorescences and *de novo* transcriptome sequencing of male and female inflorescences. Extensive sets of PCR primers belonging to different DNA fingerprinting techniques including RAPD, AFLP, SCoT, ILP, TEs, EST-SSR and gSSR and those that specific to transcripts identified from SSH and transcriptome analysis were exhaustively tested to identify sex-linked markers. Although none of the sex-linked markers was obtained from this study, we have narrowed the path towards the sex identification of Asian Palmyra palm. Because identifying sex-linked markers for this species appeared to be extremely difficult as opposed to works in other dioecious plants (Heikrujam et al., 2015), we discussed the nature of this work in conjunction with others successfully identified markers with future directions. Furthermore, this work provided the first *de novo* transcriptome sequencing of Asian Palmyra palm. Lists of candidate transcripts that are specific to sexes and developmental stages of male and female inflorescences are presented, and these will be useful for further study on sex determination, sexual development and floral development of Asian Palmyra palm.

## Materials & Methods

### Plant materials

For DNA isolation, young leaves were collected from mature palm plants with known sexes from various locations in three regions of Thailand: the southern region (Song-Khla and Surat-Thani provinces), the central region (Phachinburi, Phetchaburi, Pathum-Thani, Nakon-Pathom, Kanchanaburi, Nakhon-Sawan and Chainat provinces) and the northeastern region (Nakhon-Ratchasima, Burirum, Ubon-Ratchathani, Kalasin and Amnat-Charoen provinces). For RNA isolation, male and female inflorescences were collected from the southern region (Song Khla province) and the central region (Nakon-Pathom province).

### Nucleic acid isolation

**Figure 1:**
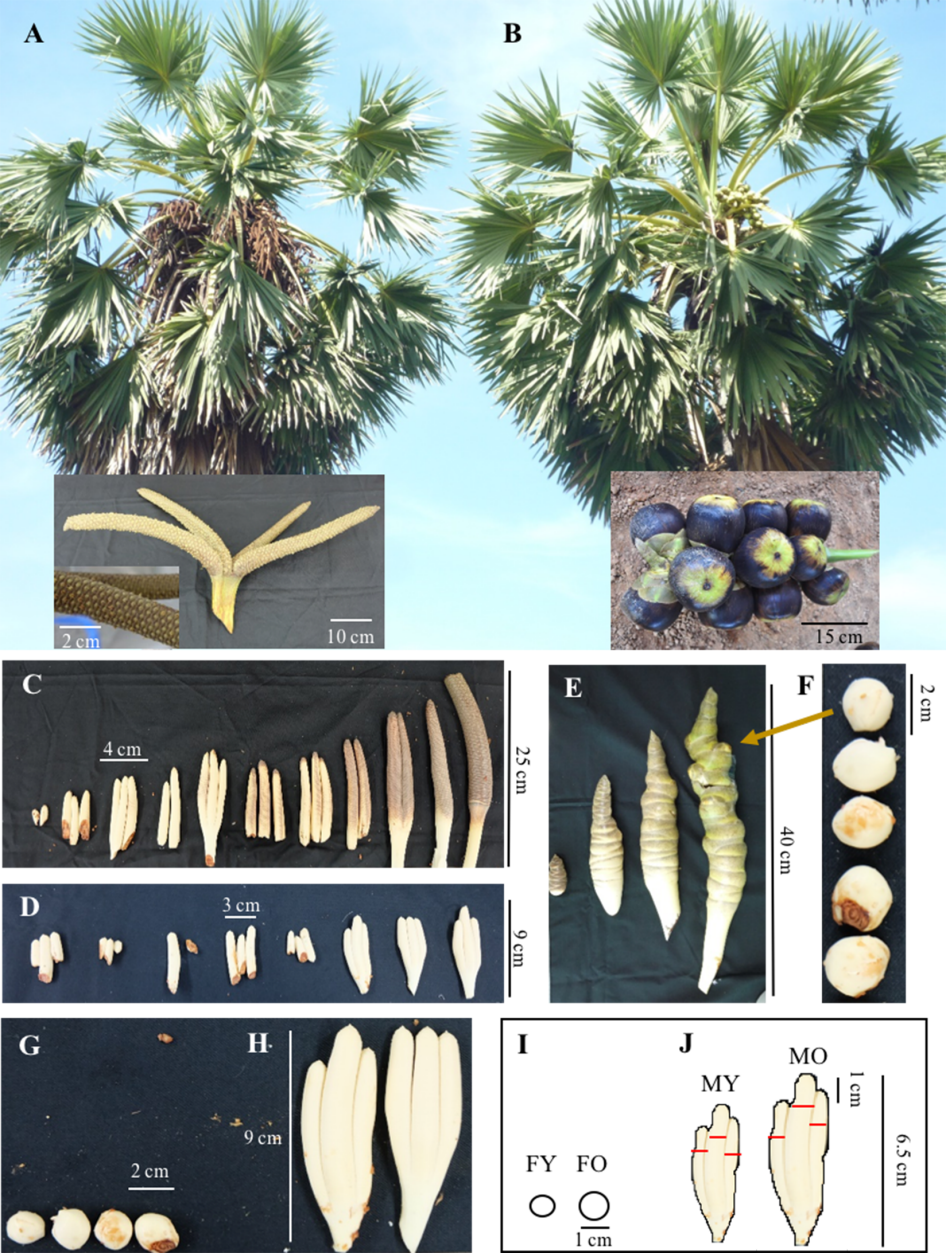
Asian Palmyra palm samples from male and female plants. (A) A mature male plant with a fully developed male inflorescence and (B) a mature female plant with a female inflorescence bearing fruits. (C) and (D) Representatives of developing male inflorescences from young to mature stages. (E) Representatives of developing female inflorescences, and (F) buds extracted from female inflorescences. (G–J) Representative female and male inflorescence samples used for the SSH experiment (G and H, respectively) and *de novo* transcriptome sequencing analysis (I and J, respectively). Scale bars are indicated within.

Total DNA was isolated from young leaves using a modified method based on the CTAB method as described previously in Pipatchartlearnwong et al. (2017a) and Pipatchartlearnwong et al. (2017b). Total RNA was isolated from young inflorescences (see Fig. 1) using the modified CTAB method. Briefly, the sample was pulverized into fine powder in liquid N_2_ by using a mortar and pestle and mixed with CTAB extraction buffer [2% (w/v) CTAB, 100 mM Tris-HCl pH.8, 20 mM EDTA and 1.4 M NaCl, 2% (w/v) polyvinylpyrrolidone-90 and 2% (v/v) β-mercaptoethanol]. RNA was then precipitated using 1/3 volume of 10 M LiCl at −20 °C for overnight and then centrifuged at 11, 750 × g at 4 °C for 30 min. Total RNA was treated with DNase I (New England BioLabs^®^Inc., Ipswich, MA, USA) at 37 °C for 30 min followed by phenol: chloroform extraction and ethanol precipitation. RNA quality and quantity were analyzed by agarose gel electrophoresis and NanoDrop (Thermo Scientific, Waltham, MA, USA).

### DNA fingerprinting

The RAPD-based OPA-06 marker was conducted according to George et al. (2007) using total DNA from 20 male and 20 female samples. AFLP analysis was performed using 26 male and 46 female samples by, firstly, restriction digestion of 250 ng total DNA using EcoRI and MseI (Thermo Fisher Scientific, USA) and adaptor ligation. And, secondly, pre-selective amplification was performed in a 25 µl reaction volume containing 2 µl of the digested DNA, 5 mM dNTPs, 40 mM MgCl2, 5 ρM of each MseI adaptor+C and EcoRI adaptor+A primers and 1 unit of *Taq* DNA polymerase (Vivantis, Malaysia) using the conditions as follows: 20 cycles of 94 °C for 30 s, 56 °C for 1 min and 72 °C for 1 min, with a final extension step at 72 °C of 5 min. The reaction was diluted 20-fold using dH_2_O before being used in a selective amplification reaction: 25 µl total volume containing 5 µl of the diluted DNA, 4 mM dNTPs, 40 mM MgCl2, 5 ρM MseI+3 and 5 ρM of EcoRI+3 primers (Table S1) and 1 unit of *Taq* DNA polymerase using a touch-down condition (12 cycles of 94 °C for 30 s, 65 °C (−0.7 °C/cycle) for 30 s and 72 °C for 1 min, followed by 23 cycles using the annealing temperature at 56 °C). SCoT was performed using cDNA from four male and four female samples. SCoT primers were based on Collard & Mackill (2009) (Table S2). The PCR reaction was performed in a 20 µl volume, which included 50 ng of DNA, 4.8 mM dNTPs, 30 mM MgCl2, 20 ρM SCoT primer and 1 unit of *Taq* polymerase (Vivantis, Malaysia) using 35 cycles of 94 °C for 1 min, 50 °C for 1 min and 72 °C for 2 min, with a final extension for 5 min. The modified SCoT method was performed by mixing SCoT primers with primers from polyA, EST-SSRs of oil palm and transposon element (TE) markers (Tables S3 and S4) (Bureau & Wessler, 1994; Takata, Kishima & Sano, 2005). ILP marker based on Ukoskit & U-thoomporn (2016) (Table S5) was performed using total DNA from three male and three female samples. Each PCR reaction were performed in a 20 µl volume containing 20 ng of DNA, 4 mM dNTPs, 75 mM MgCl2, 3.5 ρM for each primer and 0.5 unit of *Taq* polymerase (Vivantis, Selangor, Malaysia) using 35 cycles of 94 °C for 30 s, 56 °C for 1.30 min and 72 °C for 30 s, with a final extension for 5 min. EST-SSR and gSSR microsatellite markers (Table S6 and S7, based on Arabnezhad et al., 2012; Billotte et al., 2004; Elmeer et al., 2011; Pipatchartlearnwong et al., 2017a; Pipatchartlearnwong et al., 2017b) were performed using total DNA from four males and four female samples using the same PCR condition as above. PCR products for RAPD, SCoT and ILP were resolved in 2% (w/v) agarose gel electrophoresis and visualized under ultraviolet (UV) light after staining with ethidium bromide. Those for AFLP, SCoT+EST-SSR, SCoT+TEs, EST-SSR and gSSR were resolved in 6% (w/v) polyacrylamide gel electrophoresis and visualized by silver staining.

### Suppression subtractive hybridization (SSH)

Total RNA from one male and one female inflorescence samples from Song Khla province were used in the study (Figs. 1G–1H). The first-strand and double strand cDNA was synthesized from 1 µg of total RNA samples using the SMART cDNA Library Construction Kit (Clontech Laboratories Inc., Palo Alto, CA, USA) following to the manufacturer protocol. Double-strand cDNA of male and female was labeled with biotin-16 dUTP using nick translation: 100 µl total volume containing 400 ng of double-strand cDNA, 3.96 mM of dGTP, dATP and dCTP, 1.6 mM of dTTP, 2.4 µl of biotin-16 dUTP (Sigma-Aldrich), 48 µM of 5′PCR PrimerII A (5′-AGCAGTGGTATACAACGCAGAGT-3′), 10x *Taq* buffer, and 5 unit of *Taq* polymerase (Vivantis, Selangor, Malaysia) using 40 cycles of 94 °C for 15 s, 65 °C for 30 s, and 68 °C for 6 min. The biotin-16 dUTP labeled cDNA was checked for its detection signal using dot blot hybridization at least at 10^−4^ dilution. First-strand cDNA of male and female cDNA was subtracted using labeled double-strand cDNA of its opposite sex at ratio 1:3 following a method from Rebrikov et al. (2004). Subtracted samples were purified by ethanol precipitation, and double-strand cDNA was synthesized by PCR reaction containing 2 µl of subtracted cDNA, 48 µM of 5′ PCR PrimerII A, 10 mM dNTPs, 5x NEB polymerase buffer and 5 unit of Q5^®^ High-Fidelity DNA polymerase (New England Biolabs) through 21 cycles of 94 °C for 15 s, 65 °C for 30s and 68 °C for 6 min. Subtracted double-strand cDNA was purified and ligated into pGEM-Teasy (Promega). Clones were selected by blue-white colony section and screened using colony PCR for > 500 bp inserted fragment, before re-selection using dot blot hybridization I and II using probes from opposite sexes. Selected clones were sequenced and analyzed against GenBank using BLAST.

### Transcriptome analysis

Transcriptome sequencing was performed by Macrogen Inc. (Republic of Korea) using 10 µg of total RNA. Total RNA was obtained from two male (MY and MO) and two female (FY and FO) inflorescence samples from Nakon-Pathom province (Figs. 1I–1J). Briefly, cDNA libraries were constructed using the TruSeq™ RNA sample preparation kit (Illumina, USA) and sequenced on a HiSeq 2000 (Illumina, USA) with paired-end 100 bp read lengths. Initial raw reads were trimmed and filtered with FastQC (http://www.bioinformatics.babraham.ac.uk/projects/fastqc) and Trimmomatic version 0.32 (http://www.usadellab.org/cms/?page=trimmomatic) software to eliminated low-quality reads (quality score lower than 20) and remove adapters. Reads were considered as high quality if more than 70% of the bases had Phred values more than Q20. Reads produced from this study were assembled by Trinity software (version r20140717) using default parameters (Grabherr et al., 2011). For similarity search, the assembled transcripts were blasted against the NCBI non-redundant protein sequence database and TAIR database using Blast2GO with the e-value cutoff <10-10. RSEM version 1.2.15 software was used to estimate transcript abundance (Li & Dewey, 2011). The assembled sequences were analyzed for Gene Ontology (GO) and Kyoto Encyclopedia of Genes and Genomes (KEGG: Kanehisa & Goto, 2000) using Blast2GO. For differential gene expression analysis, Fastq files were aligned to the assembled transcriptome using TopHat2 alignment program (Kim et al., 2013). Transcriptome annotation file (GFF) was built using the Cufflinks program (Trapnell et al., 2010) by performing a combined assembly of four transcriptome datasets (FY, FO, MY, and MO). Transcript candidates for male and female were chosen using parameters as follows; male-highly represented transcripts [FPKM > 10 and > 0.5 for MY or MO and FPKM = 0 for both FY and FO] and [FPKM > 5 for both MY and MO and FPKM = 0 for both FY and FO] with length > 300 bp, and female-highly represented transcripts [FPKM > 10 and > 0.5 for FY or FO and FPKM = 0 for both MY and MO] and [FPKM > 5 for both FY and FO and FPKM = 0 for both MY and MO] with length > 300 bp. To identify shared transcripts among the four datasets, transcripts from each dataset with FPKM > 5 and length > 300 bp were analyzed using Venny version 2.1.0. Identified male and female specific transcripts were subjected to GO enrichment analysis using Fisher’s Exact Test with FDR cutoff = 0.01. Differentially expressed genes (DEGs) among the four transcript datasets were analyzed by Cuffdiff with FDR cutoff <0.05.

## Results

### Extensive DNA fingerprinting analysis failed to identify any sex-linked marker for Asian Palmyra palm in Thailand

Previously, George et al. (2007) had developed a RAPD based male-specific marker (OPA-06_600_) for the Asian Palmyra palm population in India. Initially, we tested this marker for sex identification in our population in Thailand using up to 20 male and 24 female individuals, but this marker was unable to confirm the sexes (Fig. S1). To identify sex-linked markers for Asian Palmyra palm in Thailand, we generated male and female DNA fingerprints using ten different DNA fingerprinting techniques as presented in Table 1. Although a number of potential sex-linked bands were obtained from SCoTs/EST-SSRs and SCoTs/TEs, after sequencing and re-testing these bands using specific primers, no sex-linked marker was obtained. Despite such extensive screening of DNA markers up to 1,204 primer pairs by the ten techniques, we did not obtain any sex-linked marker. This experiment showed that the DNA fingerprinting covered here is inadequate for identifying a sex-linked marker for Asian Palmyra palm. It also suggests that sex determination region in this species could be of small and very difficult to identify. Further attempts for this scheme in the Asian Palmyra palm should be aware of this limitation. Other means of sex identification for Asian Palmyra palm should be explored.

**Table 1:** Total markers tested for DNA fingerprints between male and female plants. Numbers of male and female samples used for each marker are indicated in brackets as M and F, respectively.

Marker	Total tested markers	Amplifiable	Polymorphic marker (loci)	Sex polymorphic loci	Sex-linked marker
RAPD	1	1 (F23:M20)	0	0	–
AFLP	36	36 (F46:M26)	13 (141 loci)	0	–
SCoTs	36	36 (F4:M4)	3	0	–
SCoTs/A	36	36 (F4:M4)	3	0	–
SCoTs (36)/EST-SSRs (3)	108	64 (F4:M4)	7 (7 loci)	2 (F8:M8)	–
SCoTs (36)/TEs (11)	396	163 (F4:M4)	21 (48 loci)	48 (F4:M4)	–
Oil palm ILPs	41	36 (F3:M3)	0	0	–
Date palm gSSRs (high PIC values)	5	2 (F4:M4)	0	0	–
Oil palm EST-SSRs	289	150 (F20:M20)	11 (17 loci)	0	–
Oil palm gSSRs	256	168 (F20:M20)	8 (12 loci)	0	–
Total	1,204	583	66 (231)	50	–

### Identification of sex-related transcripts by SSH analysis using male and female inflorescence flowers

To identify genes related to sexes, we performed SSH using total RNA isolated from male and female inflorescences of Asian Palmyra palm. Because the floral development of this species is not well defined, we collected young inflorescence stems from male and female plants as soon as they emerged from the dense leaf sheets and isolated young floral tissues for RNA isolation. Female flower buds (∼2 cm in diameter) were removed from the inflorescence (Fig. 1G), while the male inflorescences (∼9 cm in length) were used as a whole (Fig. 1H). Direct cloning of subtracted-cDNA yielded 2,574 and 9,820 clones for male and female, respectively, and, after colony-PCR screening for > 500 bp inserted-fragments, we obtained 1,288 and 4,097 clones for male and female libraries, respectively (Table 2). These selected clones were re-tested against their opposite sex using two rounds of dot blot hybridization (I and II), resulting in 81 and 63 clones for male- and female-specific libraries, respectively. These clones were subsequently sequenced and searched in GenBank using BLASTX, and 60 male and 29 female clones were found matching to non-redundant genes in the plant database (Table S8). These sequences have been deposited in GenBank (JZ977504 –JZ977592) as ESTs for male or female inflorescence flowers of Asian Palmyra palm.

**Table 2:** Clone selection of SSH between male and female.

Library	Female	Male
Direct cloning by blue-white colony selection	9,820	2,574
Fragment size > 500 bp by PCR screening	4,097	1,288
Dot blot hybridization I	498	112
Dot blot hybridization II	63	81
BlastX	29	60

Among the total 99 identified clones, 91 and 98 clones share high similarities to sequences in the nuclear genome of date palm and oil palm, respectively. As oil palm has both male and female genome sequences available, we observed that all 99 sequences identified in Asian Palmyra palm are present in both male and female genomes of oil palm, suggesting that these sequences could not be used as sex-specific markers. Nonetheless, we tested 19 selected sequences on gDNA from male and female plants by PCR using specific primers (Table S9), and these failed to identify the sexes. Although this experiment was unable to provide a sex-linked marker, the list of expressed genes during male- and female-floral development of Asian Palmyra palm could be of use for future study.

**Figure 2:**
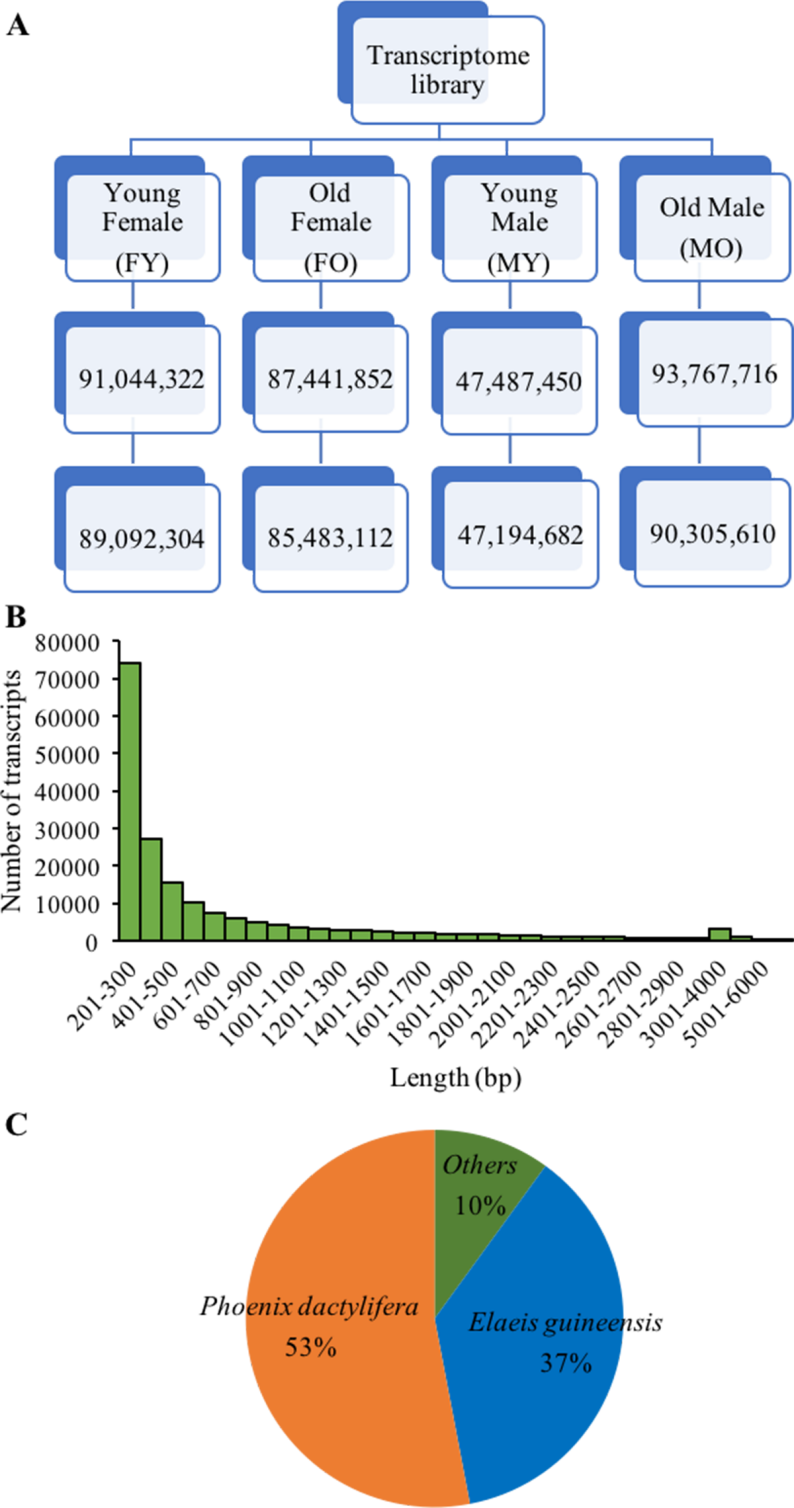
*De novo* transcriptome sequencing overview. (A) Total sequence reads (bp) after trimming and removing low quality read (<Q20) of four samples. (B) Distribution of number and length of assembled transcripts, and (C) sequence similarity of assembled transcripts to other plant species.

### *De novo* transcriptome sequencing of male and female inflorescences

To further identify sex-related genes for sex identification, we performed *de novo* transcriptome sequencing using RNA from male and female inflorescences. Four cDNA libraries were constructed from two floral stages of male and female (FY-female young inflorescences, FO-female old inflorescences, MY-male young inflorescences and MO-male old inflorescences; Figs. 1I–1J), which were in earlier stages than those used in the SSH experiment. From Illumina HiSeq2000 sequencing, we obtained 47,194,682-90,305,610 reads for each sample with an average length of 98 bp, after trimming the adapter sequences and removing low-quality nucleotides (<Q20) and short sequences (<25 nt) (Fig. 2). Sequence assembly using Trinity yielded 187,083 transcripts with 705 bp average length. Most of the assembled transcripts were between 200 and 300 bp in length, and up to 70,785 transcripts were > 500 bp in length (Fig. 2B). The transcripts were annotated by using BLASTX searches against the NCBI non-redundant protein database, and 77,578 transcripts (41.47% of initial transcripts) were identified as unique sequences with significant similarities (E <1e−10) to known protein sequences from 5,331 different species. The average alignment length (%) matched was 81.3% with the highest positive matched per alignment length being 90%, and the average percent identity was 81.24%. Most of these sequences were identified as similar sequences to those found in date palm (53%) and oil palm (37%) (Fig. 2C). The Transcriptome Shotgun Assembly are deposited at DDBJ/ENA/GenBank under the accession GFYQ00000000.

### GO classification and pathway assignment by KEGG

**Figure 3:**
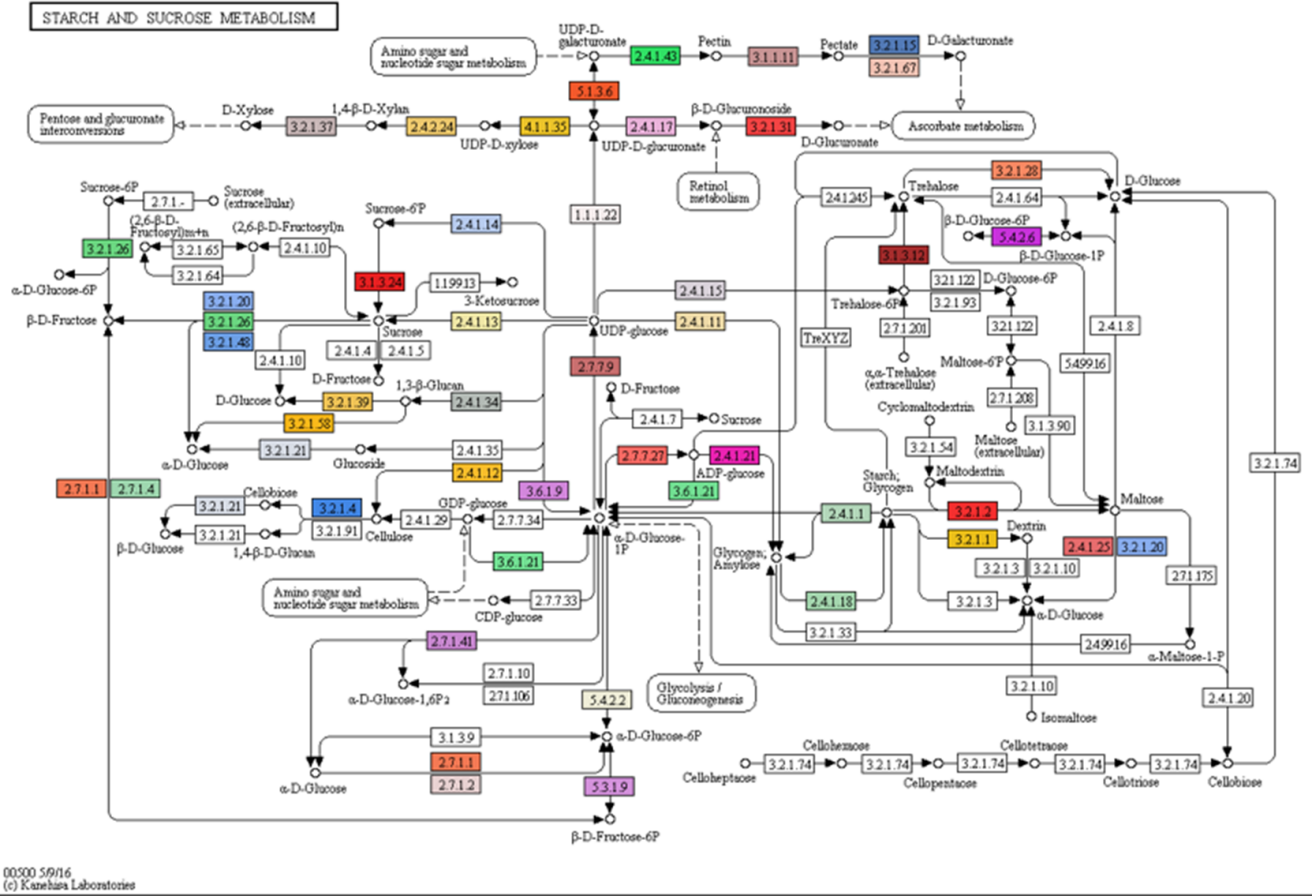
Mapping of assembled transcripts onto the starch and sucrose metabolism (map00500) taken from KEGG. Mapped enzymes are indicated by colors (see Table S10 for details). Kanehisa Laboratories 00500 5/9/16.

Gene ontology (GO) terms of the 77,578 annotated transcripts were assigned using Blast2GO program in three categories: biological process, cellular component and molecular function. The transcripts were assigned into 66 functional groups, and top GO terms with more than 1% assigned transcripts in each category are presented in Fig. S2. Binding and catalytic activity were dominant in the molecular function category, while the integral component of the membrane and nucleus dominated the cellular component category. For the biological process, oxidation–reduction process and protein phosphorylation were the most represented groups. Subsequently, function classification and pathway assignments based on Kyoto Encyclopedia of Genes and Genomes (KEGG) showed that, among the 77,578 annotated transcripts, 16,635 were annotated with enzyme code EC numbers and mapped into 139 KEGG pathways. Pathways with more than 1% matched transcripts are presented in Fig. S3. Purine (map00230) and Thymine (map00730) metabolisms were the most matched pathways with 4,913 transcripts (18.89%) and 2,581 transcripts (9.92%), followed by Biosynthesis of antibiotics (map01130) with 1,582 transcripts (6.08%). Interestingly, starch and sucrose metabolism (map00500) presented at the fourth rank with 1,187 transcripts (4.56%). Transcripts mapped into this pathway were mostly related to sugar metabolism for fructose and sucrose production, but less supported to starch biosynthesis (Fig. 3 and Table 3; see the transcript IDs in Table S10). Other related sugar metabolic pathways were also found among the list of top pathways including 1.54% of glycolysis/gluconeogenesis (map00010), 1.25% of pentose and glucuronate interconversions (map00040), 1.19% of Galactose metabolism (map00052), 1.10% of fructose and mannose metabolism (map00051), 0.87% of pentose phosphate pathway (map00030) and 0.85% of inositol phosphate metabolism (map00562). This observation coincides with the facts that both male and female inflorescences of Asian Palmyra palm produce sweet sap, which has been used for making palm sugar for centuries.

**Table 3:** The list of enzymes in starch and sucrose metabolism (KEGG map00500) identified in male and female transcripts from inflorescences of Asian Palmyra palm.

Enzyme	EC number	Number of transcripts
decarboxylase	EC:4.1.1.35	11
phosphodismutase	EC:2.7.1.41	1
endo-1,4-beta-D-glucanase	EC:3.2.1.4	56
saccharogen amylase	EC:3.2.1.2	42
glycogenase	EC:3.2.1.1	20
1,4-alpha-galacturonidase	EC:3.2.1.67	5
isomerase	EC:5.3.1.9	6
alpha-glucosidase	EC:3.2.1.48	42
1,3-beta-glucosidase	EC:3.2.1.58	6
adenylyltransferase	EC:2.7.7.27	15
maltase	EC:3.2.1.20	2
gentiobiase	EC:3.2.1.21	106
synthase	EC:2.4.1.34	39
trehalase	EC:3.2.1.28	5
invertase	EC:3.2.1.26	45
beta-glucuronide glucuronohydrolase glucuronidase	EC:3.2.1.31	6
endo-1,3-beta-D-glucosidase	EC:3.2.1.39	30
4-alpha-galacturonosyltransferase	EC:2.4.1.43	59
1,4-beta-xylosidase	EC:3.2.1.37	29
phosphorylase	EC:2.4.1.1	28
branching enzyme	EC:2.4.1.18	7
1-naphthol glucuronyltransferase	EC:2.4.1.17	33
synthase (UDP-forming)	EC:2.4.1.15	29
synthase	EC:2.4.1.14	18
synthase	EC:2.4.1.13	44
synthase (UDP-forming)	EC:2.4.1.12	98
synthase	EC:2.4.1.11	24
disproportionating enzyme	EC:2.4.1.25	8
synthase (glycosyl-transferring)	EC:2.4.1.21	13
pectin depolymerase	EC:3.2.1.15	75
diphosphatase	EC:3.6.1.9	9
glucokinase (phosphorylating)	EC:2.7.1.2	14
hexokinase type IV glucokinase	EC:2.7.1.1	44
fructokinase (phosphorylating)	EC:2.7.1.4	35
trehalose 6-phosphatase	EC:3.1.3.12	58
4-epimerase	EC:5.1.3.6	4
diphosphatase	EC:3.6.1.21	7
phosphatase	EC:3.1.3.24	6
uridylyltransferase	EC:2.7.7.9	7
pectin demethoxylase	EC:3.1.1.11	69
synthase	EC:2.4.2.24	6
(alpha-D-glucose-1,6-bisphosphate-dependent)	EC:5.4.2.2	9
beta-pgm (gene name)	EC:5.4.2.6	3
6-dehydrogenase	EC:1.1.1.22	14

### Differential expression of genes between male and female inflorescences

Among the initial 187,083 transcripts, we observed a number of transcripts that were highly represented in either male or female datasets, but none in their opposite sex (FPKM = 0); 33 and 11 transcripts were identified from female and male datasets, respectively (length > 300 bp, FPKM > 5 for both FY and FO or MY and MO, and FPKM > 10 and > 0.5 for FY or FO and MY or MO) (Table S11). Although annotations of these transcripts did not show any evidence related to genes for male- or female-specific development, we observed four cell wall-related transcripts in the female datasets, and this may reflect that the developing female flower undergoes rapid and extensive organ enlargement, whereas the male flower is limited to a small size. Because the genome sequence of Asian Palmyra palm is currently unavailable, we thus tested whether these transcripts could be used for sex identification by PCR using male and female gDNA and primers specific to these 44 transcript sequences (Table S12). However, these primers gave similar band patterns between male and female gDNA (Fig. S4), indicating that these primers cannot be used for identifying the sexes.

**Figure 4:**
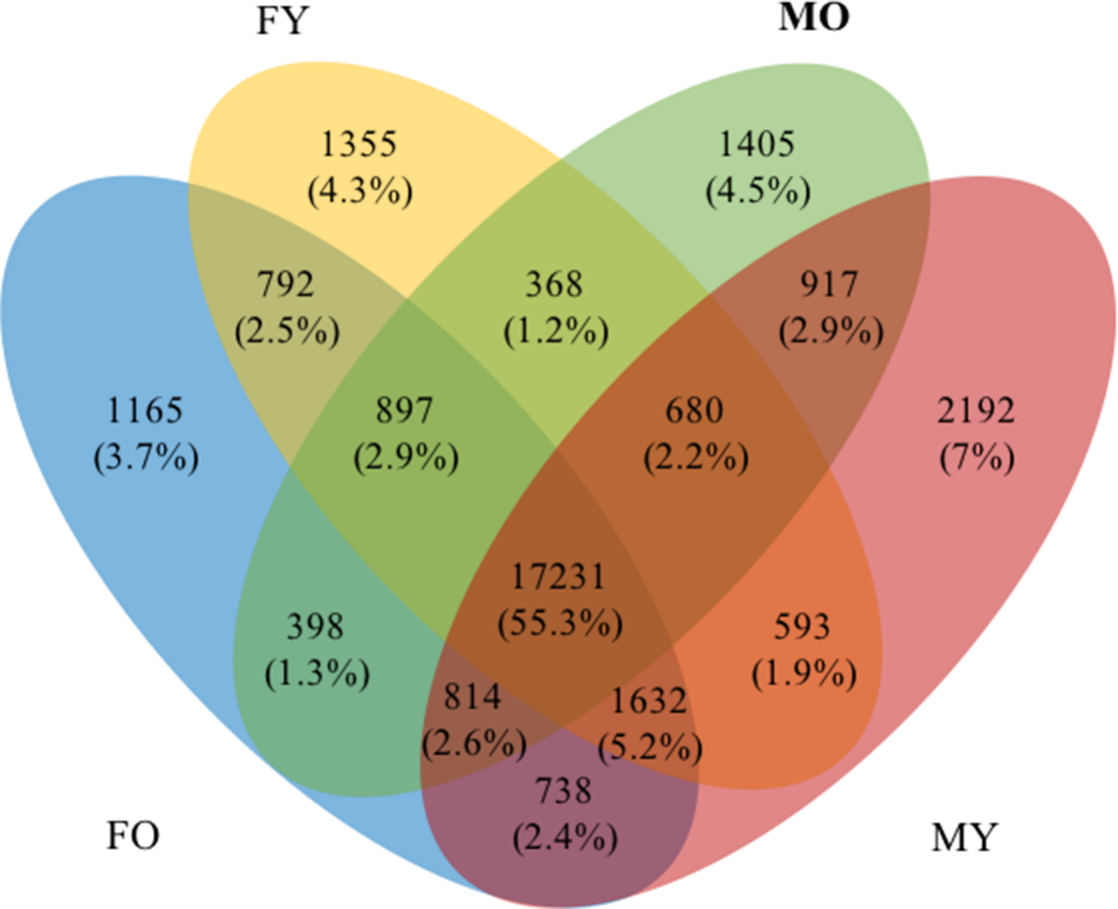
A Venn diagram of four transcript datasets. Numbers and percentages of transcripts are indicated.

By comparing the transcripts among the four datasets (length >300 bp and FPKM > 5), we found that 17,231 transcripts were shared among all datasets, and 4,514 (14.4%) and 3,312 (10.5%) transcripts were identified as male- and female-specific transcripts, respectively (Fig. 4 and Table S13). These transcripts were further classified into specific MY (2,192 transcripts), MO (1,405 transcripts), FY (1,355 transcripts) and FO (1,165 transcripts) to identify transcripts that may relate to male or female floral development stages. Subsequently, we analyzed enriched GO terms for the male- and female-specific datasets (length > 300 bp and FPKM > 5), and top enriched GO terms (FDR <1E−02) are presented in Table 4 (see Table S14 for transcript IDs). All GO terms observed here were over-represented compared to the reference sets. Interestingly, carbohydrate metabolism and cell wall-related processes were much apparent in the female-specific transcripts, while various catabolic processes for biological compounds dominated the male-specific transcripts.

**Table 4:** Enriched GO terms of male- and female-specific transcripts (FDR <1E−02).

Enriched female			Enriched male		
Go term	Cat	FDR	Go term	Cat	FDR
carbohydrate metabolic process [GO:0005975]	P	4.98E−05	L-allo-threonine aldolase activity [GO:0008732]	F	1.12E−09
phosphoribulokinase activity [GO:0008974]	F	4.98E−05	threonine aldolase activity [GO:0043876]	F	1.12E−09
cell wall organization or biogenesis [GO:0071554]	P	4.98E−05	aspartate family amino acid catabolic process [GO:0009068]	P	1.34E−08
plant-type cell wall organization or biogenesis [GO:0071669]	P	1.09E−03	threonine catabolic process [GO:0006567]	P	1.34E−08
fucose metabolic process [GO:0006004]	P	1.09E−03	glycine biosynthetic process [GO:0006545]	P	3.60E−08
cell wall biogenesis [GO:0042546]	P	1.92E−03	isovaleryl-CoA dehydrogenase activity [GO:0008470]	F	9.54E−06
plant-type secondary cell wall biogenesis [GO:0009834]	P	2.50E−03	threonine metabolic process [GO:0018927]	P	1.20E−05
plant-type cell wall biogenesis [GO:0009832]	P	2.50E−03	simple leaf morphogenesis [GO:0060776]	P	8.86E−05
cellular carbohydrate biosynthetic process [GO:0034637]	P	2.50E−03	branched-chain amino acid catabolic process [GO:0009083]	P	1.65E−04
carbohydrate biosynthetic process [GO:0016051]	P	2.50E−03	leucine catabolic process [GO:0006552]	P	1.34E−03
pyrimidine nucleoside salvage [GO:0043097]	P	3.12E−03	AMP biosynthetic process [GO:0006167]	P	1.41E−03
O-acetyltransferase activity [GO:0004026]	F	3.43E−03	AMP metabolic process [GO:0046033]	P	1.41E−03
Membrane [GO:0016020]	C	4.19E−03	methylated histone binding [GO:0035064]	F	1.41E−03
uridine kinase activity [GO:0004849]	F	4.50E−03	adenylosuccinate synthase activity [GO:0004019]	F	1.41E−03
nucleoside salvage [GO:0043174]	P	4.50E−03	PeBoW complex [GO:0070545]	C	1.41E−03
plant-type cell wall organization [GO:0009664]	P	5.70E−03	cellular amino acid catabolic process [GO:0009063]	P	1.41E−03
single-organism carbohydrate metabolic process [GO:0005975]	P	5.83E−03	phosphoacetylglucosamine mutase activity [GO:0004610]	F	1.74E−03
Endosome [GO:0005768]	C	6.68E−03	de novo’ AMP biosynthetic process [GO:0044208]	P	2.33E−03
cell wall organization [GO:0071555]	P	8.97E−03	leucine metabolic process [GO:006551]	P	3.20E−03
			geranylgeranyl-diphosphate geranylgeranyltransferase activity [GO:0016767]	F	3.26E−03
			ELL-EAF complex [GO:0032783]	C	6.81E−03
			regulation of rRNA processing [GO:2000232]	P	9.23E−03

**Notes.**

P Process F Function C Cellular

In light of this transcriptome analysis, we cross-referenced the 60 male and 29 female-specific clones from the SSH experiment to the transcript abundance data. Noting that RNA samples used in the SSH experiment were obtained from an older inflorescence stage than those used for transcriptome analysis. The transcript IDs were readily identified with more than 90% identical matches and > 300 transcript length (Table S15). However, we found that, based on FPKM values, the sex-specific clones were indeed uncorrelated to almost all of the transcript data, and only F152 (predicted proline-rich protein 4-like) clone could be identified for their expression towards the female. Though, this clone has no direct relationship to sex development based on the annotation.

To further verify sex-related genes, we analyzed differentially expressed genes among the four datasets by using Cuffdiff with FDR <0.05 cutoff. Initially, 816 transcripts were identified, but only 43 transcripts displayed differential expression between sexes with transcript length > 300 bp, FPKM > 5, GO terms and significant *q* value (<0.05) (Table 5). Among these 43 transcripts, seven transcripts were annotated with genes previously identified to be involved in sex determination and flower development: two and five transcripts for female and male datasets, respectively. Furthermore, we observed six and one transcripts encoding transcription factors that were highly expressed in female and male datasets, respectively. Although being identified for differential expression between the sexes, c1819_g1_i1 and c142400_g1_i1 transcripts encoding ethylene-responsive transcription factors were highly expressed throughout the four datasets, and these genes may be required for the floral development. The list of gene candidates indicated here could be used for a further study on sex determination and floral development in Asian Palmyra palm and other related palm species.

## Discussion

Previously, there were only two reports that attempted to develop sex identification markers in Asian Palmyra palm. George et al. (2007) presented a male-specific OPA-06_600_ marker after screening 180 RAPD primers using ten male and ten female samples from several populations in India and later used this marker to verify the sexes of more than 100 seedlings (George & Karun, 2011). Vinayagam et al. (2009) reported another attempt, but could not identify any sex-linked marker after screening up to 130 ISSR markers (with 65 polymorphic bands) based on 20 accessions from another population in India. In this work, we have also tested the OPA-06_600_ marker in Thailand populations, which were collected from the central, northeastern and southern regions, but this marker failed to confirm the sexes in our experiment. The limitation of sex-linked markers across varieties and populations is common (Heikrujam et al., 2015), and it is most likely due to sequence variation at loci used for developing the markers. Thus, it is required that sex-linked markers for populations in Thailand have to be specifically developed.

**Table 5:** Transcripts differentially expressed between sexes.

Transcript IDs	Tested samples		FPKM S1	FPKMS2	Fold change (log2)	*q* value	Blast2GO annotation
	S1	S2					
c129593_g1_i1^a^	FO	MO	7.700	63.365	3.041	0.0241	auxin-induced 15A-like (Cao et al., 2006)
c42126_g1_i1^a^	FO	MO	25.601	163.217	2.673	0.0471	glucan endo-1,3-beta-glucosidase 13 (Nagai et al., 1999)
c1819_g1_i1^a^^,^^b^	FO	MY	1738.540	99.632	−4.125	0.0383	ethylene-responsive transcription factor 4-like (Liu et al., 2008; Tao et al., 2018)
c142400_g1_i1^a^^,^^b^	FO	MY	949.073	68.455	−3.793	0.0087	ethylene-responsive transcription factor ERF017-like (Liu et al., 2008; Tao et al., 2018)
c128418_g1_i1^a^	FY	MO	6.541	146.348	4.484	0.0151	floricaula leafy homolog (Blázquez et al., 1997)
c119992_g1_i1^a^	FO	MO	4.834	59.838	3.630	0.0383	galactinol synthase 1-like (Fan et al., 2017)
c138805_g1_i2^a^	FO	MY	4.267	34.496	3.015	0.0491	argonaute 10 (Nonomura et al., 2007)
c211381_g1_i1	FY	MO	69.478	575.104	3.049	0.0241	anti-muellerian hormone type-2 receptor [*Arabidopsis thaliana*]
c140985_g1_i1	FY	MY	130.511	20.195	−2.692	0.0409	ASPARTIC PROTEASE IN GUARD CELL 2-like
c195337_g1_i1	FY	MO	44.752	547.920	3.614	0.0151	CASP 2C1
c127228_g1_i1	FY	MO	71.119	6.415	−3.471	0.0328	cellulose synthase A catalytic subunit 4 [UDP-forming]
c131838_g1_i1	FY	MY	20.253	2.104	−3.267	0.0289	cellulose synthase A catalytic subunit 9 [UDP-forming]-like
c145948_g1_i1	FY	MO	26.670	177.451	2.734	0.0448	F-box GID2-like
c124632_g1_i2	FY	MO	3.413	43.070	3.658	0.0383	high mobility group B 7 [*Nelumbo nucifera* ]
c125833_g1_i2	FO	MY	181.228	30.473	−2.572	0.0460	ingression fic1-like
c104934_g1_i1	FY	MY	5.703	70.065	3.619	0.0442	long-chain-alcohol oxidase FAO4A
c138788_g1_i1	FY	MY	483.513	19.217	−4.653	0.0133	NDR1 HIN1-Like 3-like
c106063_g1_i1	FO	MY	458.018	47.014	−3.284	0.0198	nuclease HARBI1
c181538_g1_i1	FO	MY	431.202	34.566	−3.641	0.0087	nuclease HARBI1
c134717_g2_i1	FO	MY	580.064	54.540	−3.411	0.0210	nuclease HARBI1
c126031_g2_i3	FO	MY	142.996	13.803	−3.373	0.0210	nuclease HARBI1
c126031_g2_i2	FO	MY	233.676	27.069	−3.110	0.0380	nuclease HARBI1
c116702_g1_i1	FO	MY	116.085	6.274	−4.210	0.0133	peroxidase 3-like
c129100_g1_i2	FO	MO	2.660	33.248	3.644	0.0375	probable 2-aminoethanethiol dioxygenase
c98445_g1_i1^b^	FY	MY	172.349	8.685	−4.311	0.0380	probable WRKY transcription factor 25
c129627_g1_i4^b^	FY	MO	3.978	64.185	4.012	0.0292	probable WRKY transcription factor 65
c141291_g1_i12	FO	MY	309.255	51.579	−2.584	0.0409	probable xyloglucan endotransglucosylase hydrolase 23
c141291_g1_i9	FO	MY	141.769	24.912	−2.509	0.0500	probable xyloglucan endotransglucosylase hydrolase 23
c128725_g1_i1	FO	MY	149.511	9.942	−3.911	0.0472	probable xyloglucan endotransglucosylase hydrolase 23
c105494_g1_i1	FO	MY	102.967	14.931	−2.786	0.0409	probable xyloglucan endotransglucosylase hydrolase 23
c118421_g1_i2	FY	MY	54.923	2.674	−4.361	0.0241	remorin [*Eucalyptus grandis*]
c138049_g1_i1	FY	MO	56.348	336.410	2.578	0.0472	signal [*Medicago truncatula*]
c108722_g1_i1	FY	MO	37.435	654.878	4.129	0.0472	signal [*Medicago truncatula*]
c135861_g1_i2	FY	MO	38.893	3.975	−3.290	0.0383	trans-resveratrol di-O-methyltransferase-like
c126812_g1_i2^b^	FO	MY	117.456	12.564	−3.225	0.0133	transcription factor bHLH94-like
c126812_g1_i1^b^	FO	MY	71.762	5.114	−3.811	0.0241	transcription factor bHLH94-like
c136093_g2_i1^b^	FO	MY	91.163	8.193	−3.476	0.0133	transcription factor bHLH94-like
c155603_g1_i1	FY	MY	155.137	17.684	−3.133	0.0380	U-box domain-containing 25-like
c82639_g1_i1	FY	MO	87.025	10.838	−3.005	0.0292	U-box domain-containing 26-like
c139774_g4_i1	FY	MY	192.281	28.590	−2.750	0.0472	UPF04964
c135252_g1_i1	FO	MY	40.178	2.638	−3.929	0.0198	VQ motif-containing 4-like
c137964_g3_i1	FY	MO	30.285	441.859	3.867	0.0380	Ycf68 (chloroplast) [*Carnegiea gigantea*]
c137478_g2_i2	FY	MO	50.030	369.658	2.885	0.0241	ycf68 [*Medicago truncatula*]

**Notes.**

Transcript length > 300 bp, FPKM > 5.

a involved in sex determination and flower development.

b with high FMKM values.

The success of identifying molecular markers via DNA fingerprinting may lie upon the genome size and the number of screening PCR primers, and this has been reflected in many reports for developing sex-linked markers (see Heikrujam et al., 2015 for an extensive review on the numbers of primers used in different dioecious plants). Surprisingly, date palm, which is closely related to Asian Palmyra palm (Barrett et al., 2016; Sakulsathaporn et al., 2017), readily yielded sex-linked markers when tested with only small numbers of screening primers. For example, Younis, Ismail & Soliman (2008) obtained one male-specific and two female-specific markers after screening 30 RAPD primers and five male-specific markers after screening 20 ISSR primers, Elmeer & Mattat (2012) identified 22 microsatellite loci for sex-determination in some date palm cultivars after screening 14 SSR primer pairs, and Dhawan et al. (2013) identified a male-specific marker after screening 100 RAPD primers. Because the genome size of Asian Palmyra palm has not yet been reported, it is difficult to estimate the number of screening primers to cover the genome for effective screening. Considering that our experiment used an extensive set of 1,204 primer pairs among ten techniques and there was only a single sex-linked marker identified in the previous attempts using up to 180 RAPD and 130 SSR primers (Vinayagam et al., 2009; George & Karun, 2011), this demonstrated a complexity in identifying sex-linked loci in Asian Palmyra palm.

Sex identification of date palm was subjected to a debate as newly developed sex-linked markers via DNA fingerprinting often failed when tested in other varieties or populations (Rania & Younis, 2008; Al-Mahmoud et al., 2012; Elmeer & Mattat, 2012; Dhawan et al., 2013; Maryam et al., 2016). Indeed, varieties of date palm germplasm and geographical populations were included as much when developing those markers, but the power of sex identification was limited by the variation of DNA sequences at the sex loci and, at that time, an unclear sex determination system in date palm. This problem has recently been solved by the availability of date palm genomes (Al-Dous et al., 2011), verification of sex determination loci (Cherif et al., 2013) and construction of genetic map (Mathew et al., 2014), validating that date palm has a homomorphic XY chromosome system. This sex chromosome is currently being used to develop precise sex-specific markers for specific varieties and populations of date palm (Mohei et al., 2017; Ali et al., 2018; Intha & Chaiprasart, 2018). Thus, it is potential that genome sequencing of Asian Palmyra palm could help us verify the sex determination region and, perhaps, the sex determination system in Asian Palmyra palm.

Potential sex-specific transcripts of Asian Palmyra palm were identified from SSH and *de novo* transcriptome analyses. We have tested many of these sex-specific transcripts for developing sex identification markers by PCR analysis, but none was achieved. Based on a chromosome study by Sharma & Sarkar (1956) proposing the XY chromosomes as the sex determination system of Asian Palmyra palm (XY for male and XX for female), it can be anticipated that transcripts from X chromosome should be detected in both sexes, while transcripts from the male-specific region of the Y chromosome should only be found in the male. Although most male-specific transcripts identified here are expected to be autosomal genes that support anther development or female sterility, some will be encoded by genes on the male specific region of the Y chromosome (MSY). Those male-specific transcripts can be used as markers for sex and are candidates for sex determination genes. However, PCR analysis of male-specific transcripts so far cannot identify any male-specific marker. Studying Y chromosome of Asian Palmyra palm would be essential for further development on male-specific markers.

Through the exhaustive screening of sex-specific markers using the three approaches performed in this work, this may reveal some aspects of the sex chromosome evolution of this palm species. Firstly, it is possible that the Asian Palmyra palm may have evolutionary young sex chromosomes, which recently diverged, and that the MSY is too small to be identified by the scope of this work. It could be interesting to add Asian Palmyra palm as a specimen for studying the evolution of sex chromosomes (Charlesworth, 2015). Secondly, although the work by Sharma & Sarkar (1956) had depicted clearly large difference of the sex chromosomes in the shape and size, it is still uncertain whether the XY chromosomes is the sex determination system of this palm as it has not been confirmed at molecular levels. Thirdly, because George et al. (2007) had identified a male-specific marker, which could not be used in the palm population in Thailand, there may be a unique haplotype of the MSY in Thailand. This haplotype may arise from selected individuals during migration to the southeast Asia (Pipatchartlearnwong et al., 2017a) or the sex chromosomes were recently evolved with small sex determination regions that are difficult to detect.

By comparing the SSH and transcriptome data, to our surprise, most SSH clones were uncorrelated to the sexes, given that the cDNA clones were screened through two rounds of dot blot hybridization. This observation may indicate the limitation of the SSH technique for identifying genes in a complex system. Alternatively, this may be because the RNA samples used for the SSH analysis were obtained from different flower stages from those used for the transcriptome sequencing. A potential weakness of the SSH and transcriptome sequencing was that RNA samples were isolated from developing inflorescence tissues, not from inflorescence primordia that initiate sex organs and that key sex determination transcripts may not be present or difficult to identify in the inflorescence stages (Harkess et al., 2015). Collecting the primordial tissues for this study is a very challenging task, as the Asian Palmyra palm tip is covered by many layers of thick and hard leaf sheets standing at 20 m height with no indication whether the primordial tissues will develop to be an inflorescence or a leaf, and the plant usually dies after tip removal. Moreover, the variation of sequencing depth among the samples (47–90 million reads per sample) and the lack of replicates could attribute to the complication for extracting robust conclusions from the transcriptome analysis.

Nonetheless, this work provides transcriptome data that would particularly benefit to two research areas: plant sucrose metabolism and sex development in palms and monocots. Sucrose production is one of the important areas in plant biotechnology, and many transcriptome studies have devoted to understand the control of metabolic flux towards sucrose. Most of the study was performed in major sugar crops including sugarcane (Cardoso-Silva et al., 2014; Huang et al., 2016), sugar beets (Mutasa-Göttgens et al., 2012) and sorghum ((Mizuno, Kasuga & Kawahigashi, 2016), and our data add transcript candidates for sucrose metabolism in the inflorescence of palm species. For sex development, male- and female-specific transcripts and differentially expressed transcripts between sexes were listed to provide data for further study in sex determination and male and female inflorescence development. A number of studies with similar objectives in identifying sex determination genes and underlying mechanisms of sex development in dioecious plants via the transcriptome sequencing have been reported in asparagus (Harkess et al., 2015; Li et al., 2017), *Idesia polycarpa* (Mei et al., 2017), shrub willows (*Salix suchowensis*) (Liu et al., 2013), poplar (Song et al., 2013) and *Coccinia grandis* (Mohanty et al., 2017). Most of the identified genes belong to floral development, phytohormone biosynthesis, hormone signaling and transduction, transcriptional regulation and DNA methyltransferase activity. However, with many genes playing the role during the complex developmental process, it is difficult to determine the mechanism underlying the sexual development and sex determination in dioecious species. Future progress in functional genomics addressing the identified genes would be an essential tool to solve this long-standing question.

## Conclusions

Although no sex-linked marker was obtained from exhaustive DNA fingerprinting, SSH and *de novo* transcriptome analysis, this work provides transcripts based on male and female inflorescences of the Asian Palmyra palm. Further attempts on developing sex identification markers in Asian Palmyra palm should be directed towards genomic-based approaches, particularly at the MSY. Genome analysis using SNPs have been successful in accessing sex determination loci in date palm (Al-Mahmoud et al., 2012; Ali et al., 2018) and other dioecious plant species (Zhou et al., 2018; Jia et al., 2019). Whole genome sequencing, genetic mapping, SNPs and genome-wide association study (GWAS) between male and female populations would be essential tools for further identification of sex-linked loci in Asian Palmyra palm.

##  Supplemental Information

10.7717/peerj.7268/supp-1 Fig. S1 PCR analysis of 20 male and 23 female Asian Palmyra palm samples using OPA-06_600_ RAPD marker The 600 bp ladder indicate an expected band for the male-specific marker.

10.7717/peerj.7268/supp-2 Fig. S2 GO terms of assembled transcripts Three groups of GO terms are presented including Cellular component (red bars), biological process (orange bars) and Molecular function (green bars).

10.7717/peerj.7268/supp-3 Fig. S3 KEGG and EC numbers of assembled transcripts Green bars represent percentage of transcripts categorized into different KEGG pathways, and blues bars represent number of transcripts identified to encode enzymes in different EC numbers.

10.7717/peerj.7268/supp-4 Fig. S4 PCR analysis on gDNA based on male- and female-highly represent transcripts M and F indicate male and female gDNA sample, respectively. Primers are indicated (see primer sequences in Table S12).

10.7717/peerj.7268/supp-5 Table S1 AFLP primers

10.7717/peerj.7268/supp-6 Table S2 SCoT primers

10.7717/peerj.7268/supp-7 Table S3 TE primers References of primers are included.

10.7717/peerj.7268/supp-8 Table S4 EST-SSR primers from oil palm Ta for each primer pairs is indicated.

10.7717/peerj.7268/supp-9 Table S5 ILP primers

10.7717/peerj.7268/supp-10 Table S6 SSR primers from oil palm Ta for all primer pairs was 55° C.

10.7717/peerj.7268/supp-11 Table S7 SSR primers from date palm References of primers are included.

10.7717/peerj.7268/supp-12 Table S8 Selected SSH clones Raw data. F and M represent clones from female and male, respectively. a indicates clones used for PCR analysis of gDNA from males and females.

10.7717/peerj.7268/supp-13 Table S9 SSH clone based primers for PCR analysis of gDNA from males and females

10.7717/peerj.7268/supp-14 Table S10 Transcript IDs of starch and sucrose metabolism (KEGG map00500) Transcript IDs are listed based on enzyme IDs.

10.7717/peerj.7268/supp-15 Table S11 Transcript IDs of male- and female-highly presented transcripts Female-highly presented transcripts were selected based on this criteria: (*FO* > 10*FY* > 0.5*MO* = 0*MY* = 0) + (*FO* > 0.5*FY* > 10*MO* = 0*MY* = 0) + (*FO* > 5*FY* > 5*MO* = 0*MY* = 0) +(length >300 bp), and that of male -highly presented transcripts were selected based on this criteria: (*MO* > 10*MY* > 0.5*FO* = 0*FY* = 0) + (*MO* > 0.5*MY* > 10*FO* = 0*FY* = 0) + (*MO* > 5*MY* > 5*FO* = 0*FY* = 0) +(length >300).

10.7717/peerj.7268/supp-16 Table S12 PCR primers for testing male- and female-specific transcripts Primer pairs for each transcript ID are indicated with their Tm and expected product size (bp).

10.7717/peerj.7268/supp-17 Table S13 Transcript IDs categorized based on Venn diagram among the four transcripts datasets Distinct and overlapped transcript IDs among the four transcription datasets (with FPKM > 5 and length > 300 bp) were identified and listed here.

10.7717/peerj.7268/supp-18 Table S14 Transcript IDS of enriched GO of male- and female-specific transcripts All transcripts identified with GO enrichment analysis analysis using Fisher’s Exact Test (FDR cutoff = 0.01) are presented.

10.7717/peerj.7268/supp-19 Table S15 Comparison between SSH clones and corresponding transcripts from transcriptome sequencing data Sequence identities are presented with percentage of identical matches and alignment length, and FPKM of transcripts from each transcriptome datasets are shown.

## Additional Information and Declarations

### Field Study Permissions

The following information was supplied relating to field study approvals (i.e., approving body and any reference numbers): Sample collections were conducted on privately-owned lands.

### Data Availability

The following information was supplied regarding data availability: The sequence of SSH clones are available in GenBank (JZ977504–JZ977592). The Transcriptome Shotgun Assembly are available at DDBJ/ENA/GenBank (accession GFYQ00000000). Other raw data are available in Figs. S1–S4 and Tables S1–S15.
