# Supplementary figures and images for: Towards sex identification of Asian Palmyra palm (Borassus flabellifer L.) by DNA fingerprinting, suppression subtractive hybridization and de novo transcriptome sequencing
Source: PeerJ. 2019 Jul 10;7:e7268. doi: 10.7717/peerj.7268 (PMC6626516; doi:10.7717/peerj.7268)

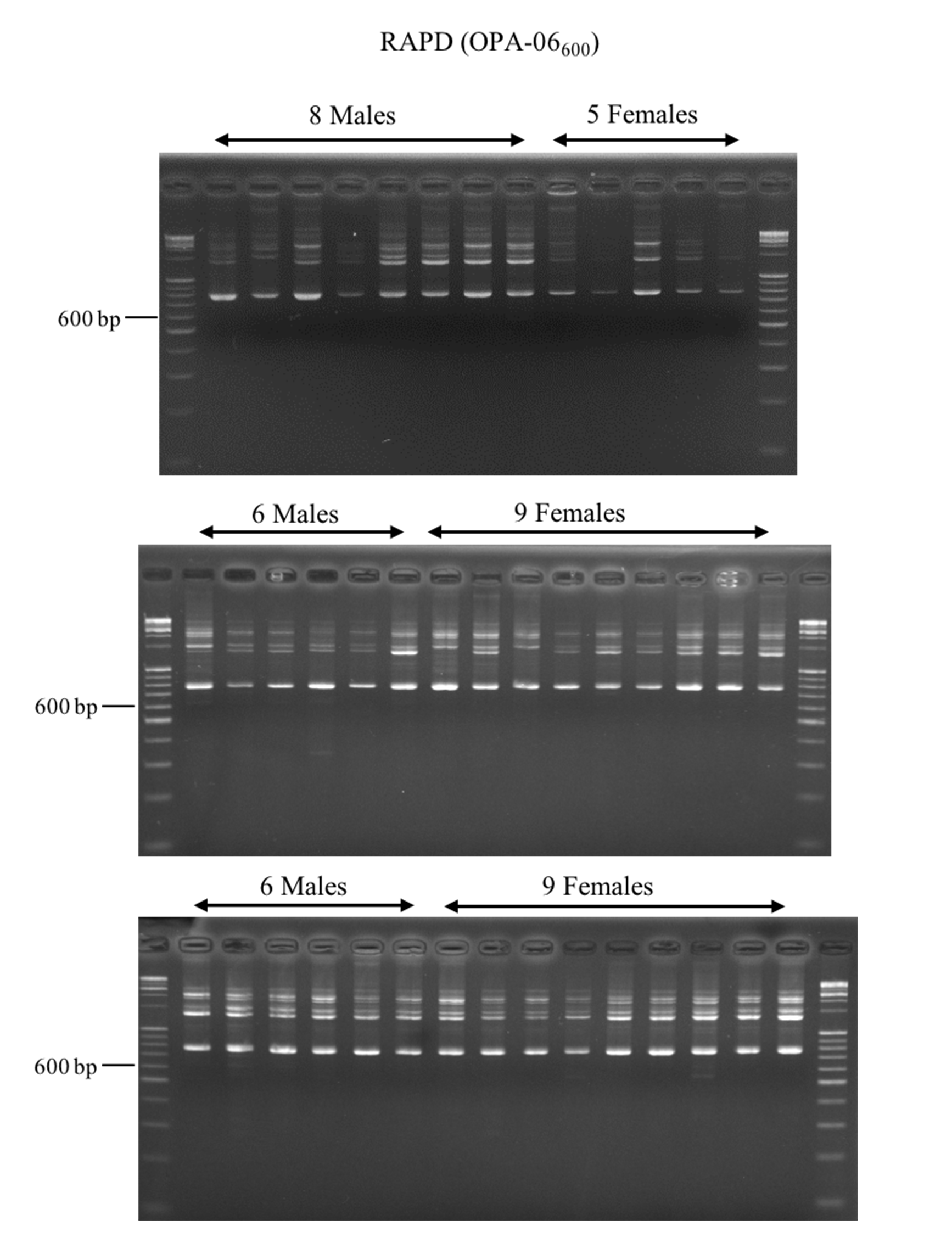

Supplement: Fig. S1 — The 600 bp ladder indicate an expected band for the male-specific marker. [file peerj-07-7268-s001.png]

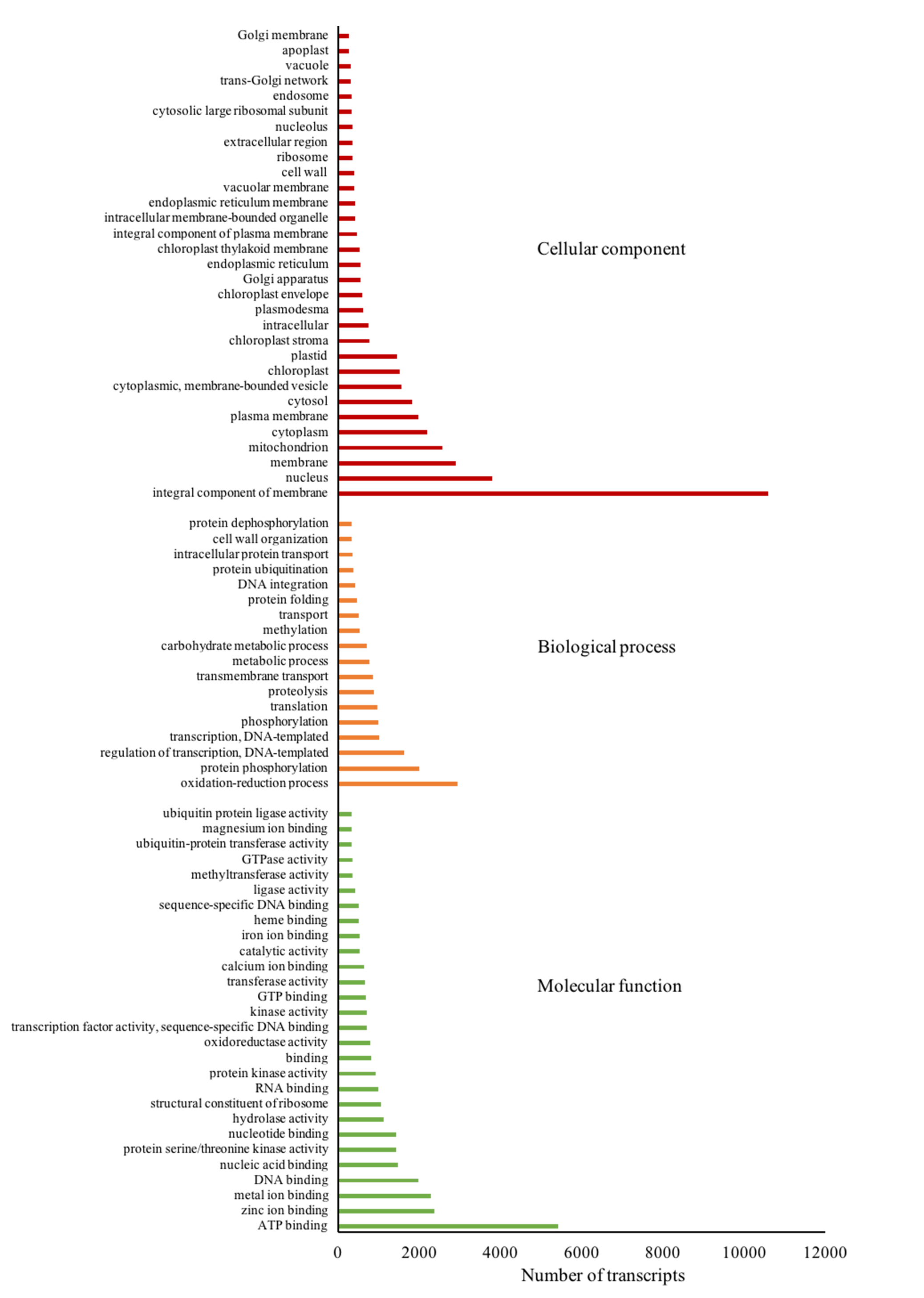

Supplement: Fig. S2 — Three groups of GO terms are presented including Cellular component (red bars), biological process (orange bars) and Molecular function (green bars). [file peerj-07-7268-s002.png]

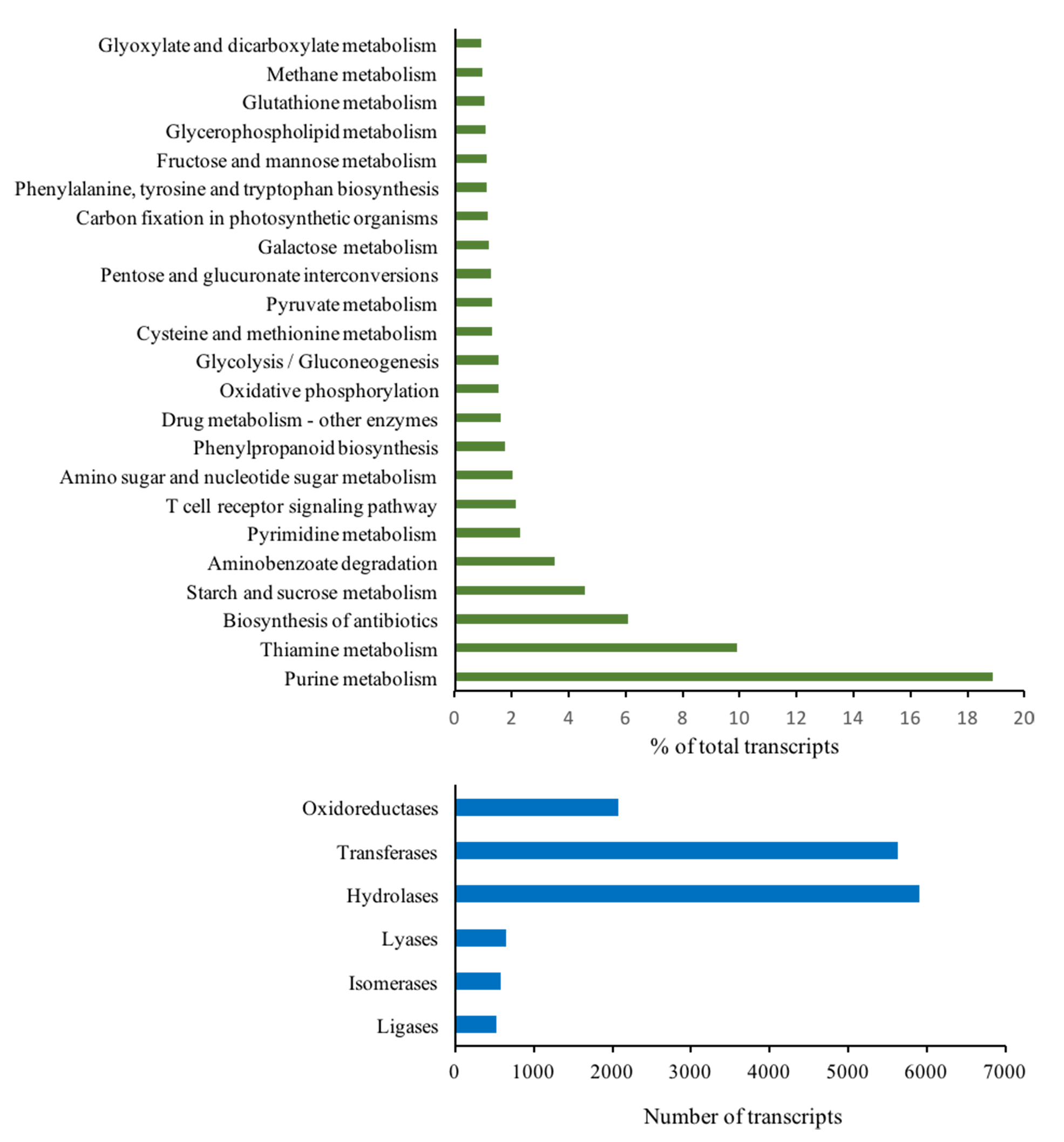

Supplement: Fig. S3 — Green bars represent percentage of transcripts categorized into different KEGG pathways, and blues bars represent number of transcripts identified to encode enzymes in different EC numbers. [file peerj-07-7268-s003.png]

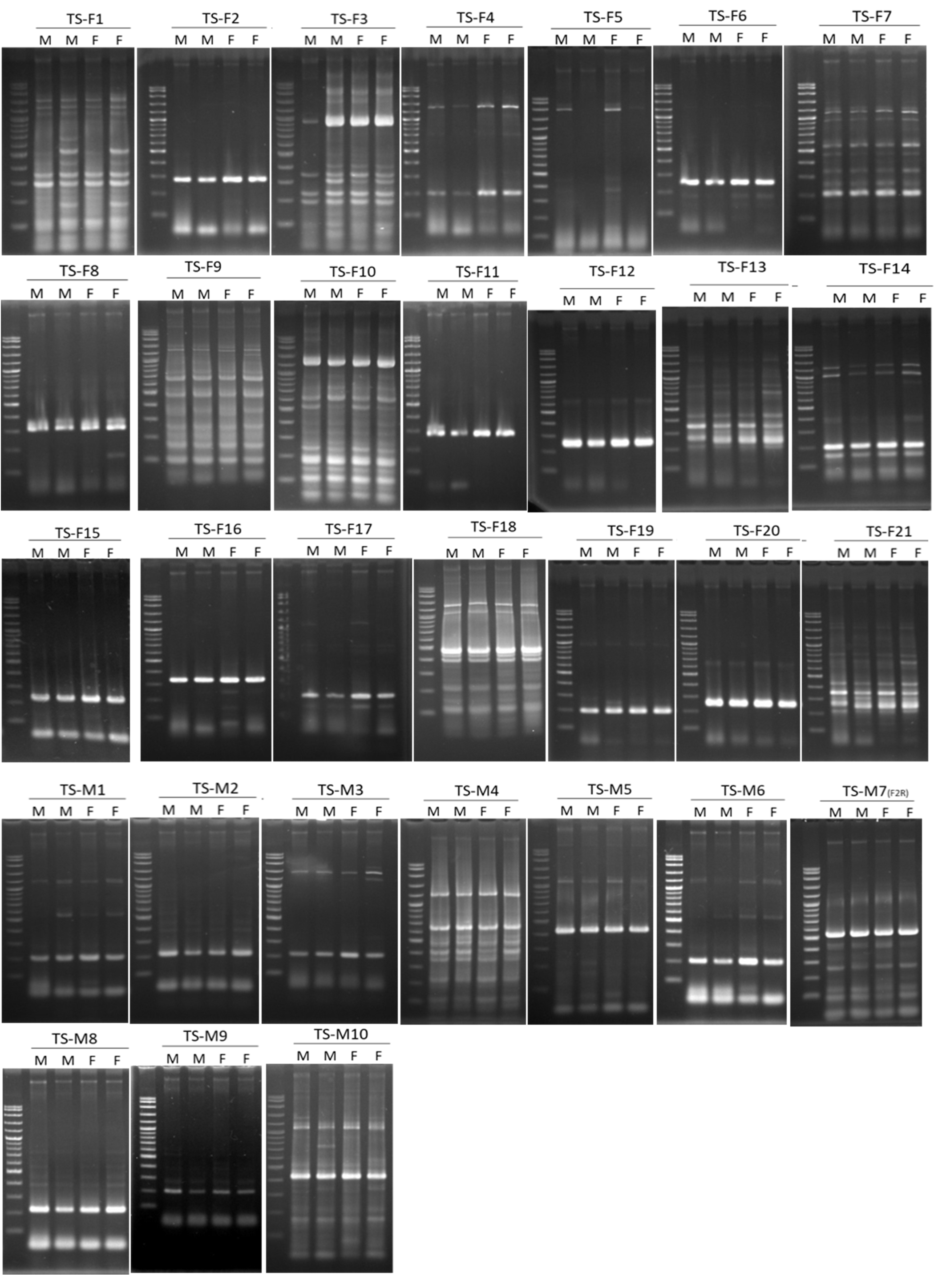

Supplement: Fig. S4 — M and F indicate male and female gDNA sample, respectively. Primers are indicated (see primer sequences in Table S12). [file peerj-07-7268-s004.png]
